# Factors associated with self-care activities among adults in the United Kingdom: a systematic review

**DOI:** 10.1186/1471-2458-9-96

**Published:** 2009-04-05

**Authors:** Angela Ryan, Sue Wilson, Aliki Taylor, Sheila Greenfield

**Affiliations:** 1 Department of Primary Care and General Practice, The University of Birmingham, Edgbaston, Birmingham, B15 2TT, UK

## Abstract

**Background:**

The Government has promoted self-care. Our aim was to review evidence about who uses self-tests and other self-care activities (over-the-counter medicine, private sector, complementary and alternative medicine (CAM), home blood pressure monitors).

**Methods:**

During April 2007, relevant bibliographic databases (Medline, Embase, Cumulative Index to Nursing and Allied Health Literature, Applied Social Sciences Index and Abstracts, PsycINFO, British Nursing Index, Allied and Complementary Medicine Database, Sociological Abstracts, International Bibliography of the Social Sciences, Arthritis and Complementary Medicine Database, Complementary and Alternative Medicine and Pain Database) were searched, and potentially relevant studies were reviewed against eligibility criteria. Studies were included if they were published during the last 15 years and identified factors, reasons or characteristics associated with a relevant activity among UK adults. Two independent reviewers used proformas to assess the quality of eligible studies.

**Results:**

206 potentially relevant papers were identified, 157 were excluded, and 49 papers related to 46 studies were included: 37 studies were, or used data from questionnaire surveys, 36 had quality scores of five or more out of 10, and 27 were about CAM. Available evidence suggests that users of CAM and over-the-counter medicine are female, middle-aged, affluent and/or educated with some measure of poor health, and that people who use the private sector are affluent and/or educated.

**Conclusion:**

People who engage in these activities are likely to be affluent. Targeted promotion may, therefore, be needed to ensure that use is equitable. People who use some activities also appear to have poorer measures of health than non-users or people attending conventional services. It is, therefore, also important to ensure that self-care is not used as a second choice for people who have not had their needs met by conventional services.

## Background

Self-care is "the care taken by individuals towards their own health and well being [1]. The Government has promoted self-care on the basis that the public favour more control over their health [1] and self-care improves health outcomes and appropriate use of health and social care services [2].

Diagnostic self-tests for over 20 conditions can be bought in pharmacies or over the internet [3]. Some provide results at home or a sample is sent to a laboratory and results are returned by post or email. Either way, people who self-test do not need to discuss why they decided to have the test or the results with a clinician. Members of the public may consider that using self-tests is self-care and, therefore, desirable, but direct access to self-tests has the potential to reduce or reinforce inequity. People who can afford a test may simply wish to check on their health, for example with a home cholesterol test, and this could free up conventional services for other people. Alternatively, people who are unable to communicate their needs to a health professional could buy expensive and perhaps undesirable tests for home use. It is, therefore, important to understand who uses self-tests so that targeted education about appropriate testing can be provided and equitable access to corresponding conventional services can be assured. Our aim was to generate hypotheses about who uses self-tests by reviewing evidence for factors that are associated with self-testing and, because of a lack of evidence about self-tests, similar activities.

## Methods

### Scope of the review

Other than self-testing, activities under review were use of over-the-counter (OTC) medicine, private care, complementary and alternative therapies (CAM), and home blood pressure (BP) monitors. These were chosen because they can be initiated without the involvement or recommendation of a conventional health professional and this is a defining feature of self-testing. Furthermore, they usually necessitate the user taking an active role, for example visiting a pharmacy, similar to buying a self-test. The review was restricted to studies about the United Kingdom (UK) because use of these activities is likely to be related to the accessibility of the health care system. The review was also restricted to studies published in the last 15 years because the availability of the activities will have altered as fashions and technologies have changed.

### Search strategy

A search strategy was designed for each activity: for example, to look for studies about self-test use, titles of papers were searched for the terms "self diagnos$" or "self test$" or "home test$" or "home diagnos$", where $ denotes truncated terms. This was then adapted for each database (table 1): for example, databases where MeSH headings are assigned to papers were searched using appropriate headings, such as "self medication" to look for studies about OTC medicine use. Searches were conducted during April 2007, but Medline was searched again in July 2008 for more recently published papers about self-testing.

**Table 1:** Number of potentially relevant papers identified and number of these papers that were eligible by activity and database^a^.

Subject	Complementary and alternative medicine (CAM)		Over-the-counter (OTC) medicine		Private care		Home blood pressure (BP) monitors		Self-tests		Total^b^	
Database	Inc.	Total	Inc.	Total	Inc.	Total	Inc.	Total	Inc.	Total	Inc.	Total
Medline	16	64	8	37	4	7	0	1	0	3	28	106
Embase	9	53	11	28	1	3	0	3	0	5	18	86
Cumulative Index to Nursing and Allied Health Literature	13	29	2	7	0	0	0	0	0	0	15	36
Applied Social Sciences Index and Abstracts	9	30	3	7	1	2	0	0	0	0	12	38
PsycINFO	8	20	3	8	1	2	0	0	0	0	12	29
British Nursing Index	4	30	0	4	0	1	0	0	0	0	4	35
Allied and Complementary Medicine Database	2	9	0	0	0	0	0	0	0	0	2	9
Sociological Abstracts	0	1	0	0	0	1	0	0	0	0	0	2
International Bibliography of the Social Sciences	1	1	0	4	0	0	0	0	0	0	1	5

References of other papers	3	3	1	1	2	2	0	0	0	0	6	6
Grey literature	1	1	0	0	0	0	1	1	0	0	2	2

Total^b^	30	135	15	62	7	13	1	4	0	5	49	206

^a^The Arthritis and Complementary Medicine Database and the Complementary and Alternative Medicine and Pain Database were also searched but no potentially relevant papers were identified.

^b^Totals may be less than the sum of the components because some papers were identified by different databases during searches related to more than one subject area: 193 papers were identified during searches related to one activity (122 related to CAM, 50 related to OTC medicine, 12 related to private care, four related to home BP monitors, and five related to self-testing) and 13 were identified during searches related to two activities (12 related to CAM and OTC medicine, and one related to CAM and private care).

Where many papers were returned, searches were refined with filters to identify appropriate study designs: for example, titles were searched for terms such as "factor$" or "characteristic$", and databases where MeSH headings are assigned were searched using headings such as "Epidemiologic Studies" or "Health Surveys". Filters to identify studies conducted in the UK were also used: the title, abstract, institution and country of publication were searched for "UK", "United Kingdom", "GB", "Britain" and the constituent countries. Finally, where possible, searches were limited to studies involving humans, written in the English language, and published from 1993 to 2007. As studies related to private care could have been published in economic as well as medical journals, The Journal of Health Economics, The Journal of Public Economics and The Economic Journal were hand-searched. References of eligible papers were also reviewed.

### Inclusion and exclusion criteria

Abstracts of potentially relevant studies were reviewed. Where it was unclear whether the study was eligible, the paper was retrieved and assessed. Studies were included if they were published during the last 15 years in a peer-reviewed journal and they reported factors, reasons or characteristics associated with a relevant activity among adults resident in the UK.

Studies were excluded if they did not concern a relevant activity or report factors, reasons or characteristics associated with an activity. Remaining studies were then excluded: if they did not involve adults or did not differentiate between children and adults; if they specified that the activity was initiated by a doctor or nurse; if they only studied intention or willingness to do an activity; if they involved people with specific conditions where the results would not be generalisable; or if they did not involve UK residents or differentiate between residents of the UK and other countries. Finally, reviews, letters or opinions were excluded, although reviews were retrieved so that relevant references could be identified.

### Quality assessment

Proformas based on tools from the Critical Appraisal Skills Programme [4,5] were used. The quantitative proforma included questions about whether the results of the study were likely to be valid, for example whether the study population was recruited appropriately (additional file 1). The qualitative proforma included questions about whether the study was likely to be rigorous and credible, for example whether the recruitment strategy was appropriate to the aims (additional file 2). Each paper was given a score out of ten by two reviewers with discrepancies resolved by discussion. Papers with scores of eight or more were termed high quality, papers with scores of more than five but less than eight were termed medium quality, and papers with scores of five or less were termed low quality. Low quality publications were included to give a complete picture and because such studies could indicate areas where higher quality research is needed. Another proforma was used to extract information from eligible papers.

## Results

Two hundred and six potentially relevant papers were identified in April 2007: 49 were eligible (table 1) and 157 were excluded (table 2). Medline identified most (n = 28) eligible papers. Embase identified the next highest number (n = 18), but there was considerable overlap with Medline: adding Embase only identified three more papers, whereas the Applied Social Sciences Index and Abstracts and PsycINFO databases both identified five more papers.

**Table 2:** Reasons why potentially relevant papers were excluded.

	Complementary and alternative medicine	Over-the-counter medicine	Private care	Home blood pressure monitors	Self-tests	Total^a^
Study did not identify factors, reasons or characteristics associated with a relevant activity (1)	21	25	3	2	4	54
Study did not involve adults or differentiate between children and adults (2)	11	5	0	0	0	14
Activity was initiated by a conventional health professional (3)	2	0	0	0	0	2
Outcome was simply the intention or willingness to do an activity (4)	1	3	0	0	0	4
Study involved a selected population (5)	56	12	2	1	0	65
Study did not relate to UK residents (6)	8	1	1	0	0	10
Review, letter or opinion (7)	6	1	0	0	1	8

Total	105	47	6	3	5	157

^a ^Totals may be less than the sum of the components because some papers were identified by different databases.

### Complementary and alternative medicine

#### Eligible papers

Thirty eligible papers were identified. Three were also identified in searches related to OTC medicine: one looked at practitioner-delivered and OTC treatments and the former results are presented in this section [6], whereas the other two did not make this distinction and all their results are discussed here [7,8]. Another paper was also identified in searches related to private care: the results are presented in this section as the study involved CAM patients [9]. This study and two others were reported across two papers [7,9-13], leaving 27 studies (additional file 3).

Eligible studies looked at different therapies, for example acupuncture [14] or alternative medicines not prescribed by a doctor [15], during different periods, for example ever [7,13] or currently [14]. One study involved interviews [16], one was a qualitative survey [17], three used questionnaires plus interviews [9,10,18,19], and one was a case note review [20]. Twenty one studies were questionnaire surveys, but only seven of them used population-based samples, for example people registered with general practices [11,12]. The remainder used selected samples, for example people visiting a practitioner [14].

One questionnaire survey had a high quality score [11,12], 18 had medium scores, and two had low scores [21,22]. Three qualitative studies had medium scores [16,17,19] and two had low scores [9,10,18].

#### Results

Using adjusted analyses, the high quality population-based survey found that being female was linked with visiting a CAM practitioner in the last three months [11,12], a medium quality population-based survey reported that taking non-prescribed alternative medicines was more likely in women than men [15], and another medium quality study found that lifetime CAM use was more likely among female than male GP attendees [7,13]. Although samples and analysis methods varied, 10 other studies reported a link between being female and CAM use [6,14,17,18,20,23-27].

Using adjusted analyses, the high quality population-based survey found that people aged 34–49 were most likely to consult a chiropractor or osteopath [11,12], and a medium quality survey found that past CAM use was more likely among GP attendees under 70 than older attendees [7,13]. Three medium quality population-based surveys used unadjusted analyses to show that use was most common among people aged 45–64 [6], 35–64 [24] and 45–54 [28], and another medium quality study described higher proportions of acupuncture patients being aged 35–64 than the general population [14]. Two medium quality surveys described that CAM users were most commonly aged 30–49 [23] and 35–44 [17], and another medium quality study described CAM patients as having a median age of about 45 [20]. A low quality population-based survey described a peak in use at age 45 [18]. Three further studies looked at age but compared people visiting CAM practitioners with GP and/or outpatient attendees [25,29,30].

Only three medium quality studies looked at ethnic origin. Using an adjusted analysis, one found that people of Black African origin were more likely than white people or people of South Asian origin to take non-prescribed alternative medicines [15]. Another reported that white students did not have a tradition of CAM use, although the analysis was limited by small numbers [19]. In contrast, the third found that most patients at a CAM hospital were white, although there was no comparison group [26].

Using population-based surveys and adjusted analyses, a high quality and a medium quality study found, respectively, that people from higher social classes were more likely to have visited a CAM practitioner in the past three months [11,12] or be taking non-prescribed alternative medicines [15] than other people. Using unadjusted analyses, two medium quality population-based surveys found that CAM use was more common among affluent than non-affluent groups [24,28], and another medium quality study reported that use was more common among GP attendees with higher incomes [7,13]. Two medium quality studies found that CAM patients had higher incomes [29] or occupational status [25] than GP patients, although again analyses were unadjusted. A further medium quality study described people visiting a Chinese medicine practitioner as having disposable income [20].

Three medium quality studies linked education and CAM use, although they all used unadjusted analyses. A population-based survey found that use was more likely among people who were older than younger when they left education [28]. Another found that use was more common among GP attendees with higher than lower educational attainment [7,13]. The third study found that CAM patients generally had a longer education than GP patients [31].

A link with poor health was supported by several studies. Using adjusted analyses, the high quality population-based survey found that people with a long-standing illness or who saw their GP more often were more likely to have seen a CAM practitioner recently than other people [11,12], and a medium quality study found that CAM patients were more likely than GP patients to have psychiatric morbidity [29]. Using unadjusted analyses, a low quality population-based survey found that CAM users had higher GP attendance rates and were more likely to have severe or chronic conditions than non-users [18], and two medium quality studies found that CAM patients were more likely than GP patients to have had a serious illness [30], chronic illness [30] and longer illness [30,32]. Two medium quality studies also looked at symptom length: one described most Chinese medicine patients as having symptoms for over a year [20], and the other described new CAM patients as having longer symptoms than GP patients, although CAM patients also had lower pain scores [31].

Some studies described reasons for using CAM, for example an acquaintance's recommendation [9,10,33] or because a family member had done so [8]. Others cited users' views about orthodox medicine's disadvantages, for example rushed appointments [9,10] and its limited effectiveness [9,10,16,27], compared with CAM's attractions, for example its effectiveness [27] and sensitive practitioners with time to listen [30].

The high quality population-based survey reported that non-smokers and people who took regular exercise were more likely to have seen a chiropractor or osteopath than other people [11,12]. Two medium quality studies with adjusted analyses also reported a link with healthy living: compared to GP patients, CAM patients had healthier lifestyles [30] and were more likely to believe in healthy living [29]. Similar to this, a low quality unadjusted survey found that patients at alternative therapy centres were more likely to be health conscious and know about health than health centre attendees [21]. A medium [29] and a low quality study [21] also reported that CAM users were more likely to believe that they controlled their health or less likely to believe that doctors controlled their health than users of orthodox medicine.

### Over-the-counter medicine

#### Eligible studies

Fifteen eligible papers relating to 15 studies were identified. Two are discussed in the CAM section [7,8], leaving 13 studies (additional file 4). The studies looked at use or purchase of any or specific medicines, such as analgesics [34]. Two were qualitative studies [35,36] and 11 were surveys. Four surveys used population-based samples [6,34,37,38], for example from health authority registers. One survey sampled people who were shopping [39] and two sampled people attending general practices [40,41]. The remaining four surveys looked at OTC purchasers without a comparison group [42-45]. Four surveys [34,37,38,40] had high quality scores. Three surveys [6,39,41] and the two qualitative studies [35,36] had medium scores. The other four surveys had low scores [42-45].

#### Results

Two low quality surveys described the sex of purchasers of OTC medicines and found that most were female [44,45]. Four population-based surveys agreed that use or purchase was more common in females: three were high quality and used adjusted analyses [34,37,38] and one was medium quality but unadjusted [6]. Using unadjusted analyses, a high quality survey of GP attendees [40] and a medium quality survey of shoppers [39] also found that being female was linked with using or purchasing OTC medicines.

One high quality population-based survey reported on ethnicity: this found that people who were white were more likely to use herbal supplements than other people [37]. Most studies looked at age. Three high quality population-based surveys found, using adjusted analyses, that purchase or use of OTC medicines was more common in people aged 35–44 [38], 45–64 [37], and under 60 [34]. One medium quality adjusted survey found that GP attendees [41] aged under 60 were more likely to use OTC medicines than older people, and another medium quality unadjusted survey reported a similar result for shoppers [39]. Using unadjusted analyses, a high quality survey of GP attendees [40] and a medium quality population-based survey [6] found that use or purchase of OTC medicines was highest in the 45–64 age group, and two low quality surveys reported that most OTC buyers were 46–60 [44] or 36–45 years [45].

Three high quality population-based surveys [34,37,38], one high quality survey of GP attendees [40] and one medium quality survey of GP attendees [41] found that OTC medicine use or purchase was associated with affluence. The studies used different measures though, for example occupation [38] or the Carstairs deprivation category [34], and only two found the association after adjusting for other variables [37,38]. One of the high quality surveys also reported an association with educational attainment, but only education remained significant in the adjusted analysis [34]. This could suggest that the association with affluence may be related to education, but there was also a link with paying for prescriptions in the adjusted analysis and this could be a surrogate for affluence. A low quality but adjusted survey of pharmacy customers also found an association with paying for prescriptions [43], and two medium quality unadjusted analyses of GP attendees [41] and shoppers [39] reported a similar link.

Only three studies found a link between purchase or use of OTC medicines and poor health, but all were high quality population-based studies with adjusted analyses. Two studies looked at self-reported health [34,38] and the third looked at psychiatric morbidity [37].

Two high quality population-based surveys examined behaviour. One found that herbal supplement use was associated with not smoking and being active, although only being active remained significant after adjusting for other variables [37]. This suggests that use may be associated with healthy behaviours. The other survey found an unadjusted association between non-prescription analgesics and drinking alcohol, but the comparison was with non-drinkers so this is probably not a healthy behaviour [34].

Some low quality surveys described reasons for OTC use, for example a prompt by an acquaintance [45], habit [44], homeopathic remedies being more natural [45], or symptoms not being severe enough for the doctor [42]. Some of these reasons were echoed in the qualitative studies, for example experience and influence of family members [35], doctors being unable to help with some problems [36], and prescription medicines being chemical [36].

### Private sector

#### Eligible studies

Seven eligible papers were identified. One was also identified in searches related to CAM and is discussed in the CAM section [9]. The remaining six papers related to six studies (additional file 5). Five studies using data collected during surveys to look at determinants of insurance [46-48] and who actually uses private care [49,50]. The sixth study used data from general practice records to look at who was most likely to be privately referred [51]. The study that used general practice data had a high quality score [51], three studies had medium scores [47,48,50], and two had low scores [46,49].

#### Results

One medium quality study, using an adjusted analysis, reported that women were less likely to have private insurance than men [47]. A link with age was found in four studies. The high quality study used an adjusted analysis to show that people aged 45–54 were most likely to be privately referred [51]. Another medium quality study, also using an adjusted analysis, found that private insurance increased with age but fell for older people [48]. A low quality study reported the same result [46], although it was unclear if the analysis was adjusted. In contrast, a medium quality study, using an adjusted analysis, found that private insurance increased with age [47].

Private care or insurance was positively associated with affluence and/or negatively associated with deprivation in all the studies. The high quality study [51] and two medium quality studies [47,48] used adjusted analyses, whereas the other medium quality study used an unadjusted analysis [50] and it was unclear if the two low quality studies had adjusted for other variables [46,49]. The measures used, for example income [46] or the Index of Multiple Deprivation [51], also varied.

Being privately insured was found to be more likely among people with than without a basic qualification in a medium quality study that used an adjusted analysis including the possible confounders of income and occupation [47]. A similar link was found in a low quality study, but it was unclear whether the analysis was adjusted [46].

Only one study reported on the relationship with health status [49]. This reported a negative association between private care and being limited in one's daily activities, but the study was low quality and it was unclear if the analysis was adjusted.

### Home blood pressure monitors

One eligible medium quality survey of 5545 people registered with general practices (54% response rate: 153 questionnaires undelivered and 2925 completed) was identified [52]. Being retired (p < 0.05), being not in employment (p < 0.05), having a long-term illness (p < 0.001) and/or not good health (p < 0.05), being a non-smoker (p < 0.01), and having used other self-tests (p < 0.0001) were linked with self-testing for high BP but only in a univariate analysis. Increasing age (p < 0.0001), being female (p < 0.0001), having a degree (p < 0.05) and living in a more affluent area (p < 0.05) remained significant after adjusting for other variables.

### Self-tests

One eligible paper about self-testing was identified in July 2008 [53]. This presented results for self-tests related to cancer from the survey of 5545 adults that had been identified in searches related to home BP monitors [52]. Using an adjusted analysis, the study found that predictors of use of a prostate specific antigen test were being male (p < 0.01), white ethnicity (p < 0.01) and older age (p < 0.01), and that use was lower among relatively deprived people (quartile 3 of the Index of Multiple Deprivation, p < 0.001). In contrast, use of a haematuria test was predicted by being relatively deprived (p < 0.05), as well as giving one's employment status as looking after the home and/or family (p < 0.01).

## Discussion

### Main findings of this study

This study reviewed evidence for factors that are associated with self-testing and similar activities among UK adults. Forty nine eligible papers were identified. Most (n = 28) were identified by Medline and most (n = 30) were related to CAM. There were 46 unique studies and most (n = 36) had high or medium quality scores. Most (n = 37) studies were, or used data from established questionnaire surveys, but only 16 of them were population-based. Survey analysis methods varied from simple descriptive to adjusted analyses, but studies generally looked at five areas – demographic factors (sex and age), affluence/deprivation, education, health status, and health behaviours and beliefs.

Being female was associated with the activity in 13 of 27 studies about CAM, eight of 13 studies about OTC medicine, and the only study about home BP monitors. Ten studies about CAM, nine studies about OTC medicine, and three studies about the private sector reported that people in the 30 to 64 year age range were most likely to undertake the activity or that use declined after 60 or 70 years. One study, however, found that private insurance was more common in men than women and as age increased [47], and the study about home BP monitors reported that use became more likely as age increased [52].

A link with affluence was presented in eight studies about CAM, five studies about OTC medicine, all six studies about the private sector, and the study about home BP monitors. Higher educational attainment was associated with use in three studies about CAM, one study about OTC medicine, two studies about the private sector and the study about home BP monitors. Education and affluence are likely to be associated though and many studies used unadjusted analyses or looked at education or affluence. Even so, no study found that the activity was less common in more affluent or educated people.

Six studies about CAM, three studies about OTC medicine, and the study about home BP monitors suggested a link with various measures of poor health, but the analyses were often unadjusted. Conflicting evidence was also presented by two studies: one found that CAM patients had longer symptoms than GP patients but also lower pain scores [31], and the other reported that private care was less likely among people limited in their daily activities than among those not limited [49].

Four studies about CAM and one about OTC medicine suggested a link with healthy lifestyles or being health conscious and knowledgeable about health, and no studies contradicted this. Two studies also found that CAM users were more likely to believe that they control their health or less likely to believe that doctors control their health than users of orthodox medicine.

### What is already known on this topic

The aim of this study was to review evidence about who uses self-tests among UK adults. No relevant studies about self-testing were initially identified though. As a result, the scope was widened to look at similar activities, and this study is the first to draw together evidence from different areas about common factors that are associated with self-care activities.

### What this study adds

People who use CAM and OTC medicine appear similar. There is general consensus that they are usually female, middle-aged and affluent and/or educated. There is also fairly general agreement that people who undertake these activities have some measure of poor health. People who use home BP monitors appear similar to people who use CAM and OTC medicine except that use was associated with increasing age. The study about home BP monitors did not ask about hypertension though, and rising use could be related to the increased prevalence of this condition with age, perhaps unlike other conditions for which CAM and OTC medicines are used.

Similar to CAM, OTC medicines and home BP monitors, people who use the private sector appear to be affluent and/or educated. In contrast to users of other activities though, there is some evidence that males tend to have private insurance and people with good health tend to have private care. This suggests that it may not be appropriate to group studies about the private sector with studies about other self-care activities.

### Limitations of this study

Only studies conducted in the UK were included because it was felt that the health care environment would have affected choices about the activities under consideration. The review may not, therefore, be applicable to other settings.

As there was no clear equivalent activity, studies about activities with similarities to self-testing were reviewed. Each activity, however, also has differences to self-testing, for example CAM is generally considered to be outside conventional medicine, whereas self-tests could be considered an extension of orthodox medicine. There were also differences between the activities, and grouping together results from such studies may not be appropriate, for example people who use the private sector may be different to people who use the other activities. As only one study about home BP monitors and self-tests was identified, it is probably not appropriate to draw firm conclusions about users of these activities.

Self-tests were defined for this study as tests that are bought and used without involving a doctor, nurse or other conventional health professional. As a result, studies were excluded where there was evidence that the activity was always initiated by a conventional health professional. Activities could also be simply recommended by health professionals though, and this was often not asked about or, if asked about, used to group people. It was not possible, therefore, to determine whether people who used an activity after a conventional professional's recommendation were different to people who used them without any such recommendation. Similar to this, use of private care and some CAM facilities may require referral from a conventional health professional, but the idea may have come from the patient. Again though, this was often not reported or used to group people. This may be important as someone who is affluent and/or educated may be more able to influence a GP to arrange a referral, which could, at least, partly explain the link between affluence and the use of these activities.

Eligible studies often defined use in different ways. Identified studies also used different data collection and analysis methods. Questionnaire surveys were often not population-based or did not have a relevant comparison group so descriptive analyses were presented about the group using the activity. Even where there was a comparison, analyses were often not adjusted for confounding variables so it was difficult to see if associations, such as education and affluence, were independent. Factors examined also varied widely, even though more basic characteristics, such as ethnic group, were infrequently studied. It was also sometimes difficult to tell whether papers related to the same study, for example three papers used similar methods and the same authors, but the number of participants varied [27,30,32]. These issues meant that it was not possible to formally pool analyses from studies within each area.

### Future research

The government is encouraging self-care because of evidence that this will improve health outcomes and appropriate use of conventional services. The promotion of self-care may mean that self-tests are seen as desirable. It will be important, therefore, given the potential disadvantages of self-tests, to assess the impact of this policy on their use. There is a lack of evidence about who currently uses self-tests and why they use them though and a need for research in this area. It seemed sensible to collate evidence from studies about similar activities as a starting point, but it remains important, particularly given the potential disadvantages of self-tests, to use this knowledge to directly study who is using self-tests and why they are being used. The evidence from this review will, therefore, be used to design a questionnaire to look at factors that are associated with using self-tests. This review will also be useful to policy makers wishing to consider how best to promote general self-care activities by highlighting those groups who are not engaged in such activities. Further qualitative research among these groups about why self-care is not used and how this could be facilitated would be useful.

## Conclusion

This review suggests that people who engaged in self-care activities were likely to be affluent and/or educated. Self-care is, therefore, likely to require targeted promotion to ensure that use is equitable.

People who have used some self-care activities may also have poorer health than non-users or people attending conventional services. It seems reasonable that people would use self-care activities when they feel unwell, but it is important to ensure that self-care is not a second choice for people who have been dissatisfied with, or not had their needs met by conventional services. There is weak evidence that dissatisfaction with orthodox medicine was a factor in some people's decision to use CAM and that some people used OTC medicine because their doctor was unable to help them. This requires further investigation as part of the evaluation of the promotion of self-care.

## Abbreviations

OTC: over-the-counter; CAM: complementary and alternative therapies; BP: blood pressure; UK: United Kingdom.

## Competing interests

The authors declare that they have no competing interests.

## Authors' contributions

AR, SW and SG were responsible for the overall design of the study and designed the search strategy. AR conducted the searches, retrieved the papers and reviewed the literature. AR and AT or SW independently reviewed the quality of eligible papers. AR prepared the report with input from all authors.

## Pre-publication history

The pre-publication history for this paper can be accessed here:



## Supplementary Material

Additional file 1 **Box 1**. Proforma based on tools from the Critical Appraisal Skills Programme [4,5] that was used to assess the quality of eligible quantitative studies. Click here for file

Additional file 2 **Box 2**. Proforma based on tools from the Critical Appraisal Skills Programme [4,5] that was used to assess the quality of eligible qualitative studies. Click here for file

Additional file 3 **Table 3**. Summary of eligible studies related to use of complementary and alternative therapy (CAM). Click here for file

Additional file 4 **Table 4**. Summary of eligible studies related to use of over-the-counter (OTC) medicine Click here for file

Additional file 5 **Table 5**. Summary of eligible studies related to use of the private sector. Click here for file

## References

[B1] Department of Health (2005). Self Care – A Real Choice. Self Care Support – A Practical Option. London.

[B2] Department of Health (2007). Research evidence on the effectiveness of self-care support. London.

[B3] Ryan A, Wilson S, Greenfield S, Clifford S, McManus RJ, Pattison HM (2006). Range of self-tests available to buy in the United Kingdom: an Internet survey. Journal of Public Health.

[B4] Critical Appraisal Skills Programme Making sense of evidence: 12 questions to help you make sense of a cohort study. http://www.phru.nhs.uk/Doc_Links/cohort%2012%20questions.pdf.

[B5] Critical Appraisal Skills Programme (CASP) Making sense of evidence: 12 questions to help you make sense of a qualitative research. http://www.phru.nhs.uk/Doc_Links/Qualitative%20Appraisal%20Tool.pdf.

[B6] Thomas KJ, Nicholl JP, Coleman P (2001). Use and expenditure on complementary medicine in England: a population based survey. Complementary Therapies in Medicine.

[B7] Featherstone C, Godden D, Selvaraji, Emslie M, Took-Zozaya M (2003). Characteristics associated with reported CAM use in patients attending six GP practices in the Tayside and Grampian regions of Scotland: a survey. Complementary Therapies in Medicine.

[B8] Greenfield SM, Innes MA, Allan TF, Wearn AM (2002). First year medical students' perceptions and use of complementary and alternative medicine. Complementary Therapies in Medicine.

[B9] Andrews GJ (2002). Private complementary medicine and older people: service use and user empowerment. Ageing and Society.

[B10] Andrews GJ (2003). Placing the consumption of private complementary medicine: everyday geographies of older peoples' use. Health and Place.

[B11] Ong C, Petersen S, Bodeker GC, Stewart-Brown S (2002). Health Status of People Using Complementary and Alternative Medical Practitioner Services in 4 English Counties. American Journal of Public Health.

[B12] Ong C, Doll H, Bodeker G, Stewart-Brown S (2004). Use of osteopathic or chiropractic services among people with back pain: a UK population survey. Health and Social Care in the Community.

[B13] Featherstone C, Godden D, Gault C, Emslie M, Took-Zozaya M (2003). Prevalence Study of Concurrent Use of Complementary and Alternative Medicine in Patients Attending Primary Care Services in Scotland. American Journal of Public Health.

[B14] MacPherson H, Sinclair-Lian N, Thomas K (2006). Patients seeking care from acupuncture practitioners in the UK: A national survey. Complementary Therapies in Medicine.

[B15] Cappuccio FP, Duneclift SM, Atkinson RW, Cook DG (2001). Use of Alternative Medicines in a Multi-Ethnic Population. Ethn Dis.

[B16] Cartwright T, Torr R (2005). Making sense of illness: The Experiences of Users of Complementary Medicine. Journal of Health Psychology.

[B17] Richardson J (2004). What Patients Expect From Complementary Therapy: A Qualitative Study. American Journal of Public Health.

[B18] Murray J, Shepherd S (1993). Alternative or additional medicine? An exploratory study in general practice. Social Science and Medicine.

[B19] Freymann H, Rennie T, Bates I, Nebel S, Heinrich M (2006). Knowledge and use of complementary and alternative medicine among British undergraduate pharmacy students. Pharmacy World and Science.

[B20] Wadlow G, Peringer E (1996). Retrospective survey of patients of practitioners of traditional Chinese acupuncture in the UK. Complementary Therapies in Medicine.

[B21] Furnham A, Forey J (1994). The attitudes, behaviors and beliefs of patients of conventional vs. complementary (alternative) medicine. Journal of Clinical Psychology.

[B22] Furnham A (2000). Does experience of the 'occult' predict use of complementary medicine? Experience of, and beliefs about, both complementary medicine and ways of telling the future. Complementary Therapies in Medicine.

[B23] Emslie MJ, Campbell MK, Walker KA (2002). Changes in public awareness of, attitudes to, and use of complementary therapy in North East Scotland: surveys in 1993 and 1999. Complementary Therapies in Medicine.

[B24] Ernst E, White A (2000). The BBC survey of complementary medicine use in the UK. Complementary Therapies in Medicine.

[B25] Furnham A, Beard R (1995). Health, just world beliefs and coping style preferences in patients of complementary and orthodox medicine. Social Science and Medicine.

[B26] Sharples FMC, van Haselen R, Fisher P (2003). NHS patients' perspective on complementary medicine: a survey. Complementary Therapies in Medicine.

[B27] Vincent C, Furnham A (1996). Why do patients turn to complementary medicine? An empirical study. British Journal of Clinical Psychology.

[B28] Thomas K, Coleman P (2004). Use of complementary or alternative medicine in a general population in Great Britain. Results from the National Omnibus survey. Journal of Public Health.

[B29] Furnham A, Bhagrath R (1993). A comparison of health beliefs and behaviours of clients of orthodox and complementary medicine. British Journal of Clinical Psychology.

[B30] Furnham A, Vincent C, Wood R (1995). The Health Beliefs and Behaviors of Three Groups of Complementary Medicine and a General Practice Group of Patients. The Journal of Alternative and Complementary Medicine.

[B31] Paterson C (1997). Complementary practitioners as part of the primary health care team: consulting patterns, patient characteristics and patient outcomes. Family Practice.

[B32] Vincent C, Furnham A, Willsmore M (1995). The perceived efficacy of complementary and orthodox medicine in complementary and general practice patients. Health Education Research.

[B33] Emslie M, Campbell M, Walker K (1996). Complementary therapies in a local healthcare setting. Part 1: Is there real public demand?. Complementary therapies in Medicine.

[B34] Porteous T, Bond C, Hannaford P, Sinclair H (2005). How and why are non-prescription analgesics used in Scotland?. Family Practice.

[B35] Gray NJ, Cantrill JA, Noyce PR (2002). 'Health repertories': an understanding of lay management of minor ailments. Patient Education and Counseling.

[B36] Vickers KA, Jolly KB, Greenfield SM (2006). Herbal medicine: women's views, knowledge and interaction with doctors: a qualitative study. BMC Complementary and Alternative Medicine.

[B37] Harrison RA, Holt D, Pattison DJ, Elton PJ (2004). Who and How Many People Are Taking Herbal Supplements? A Survey of 21 923 Adults. International Journal for Vitamin and Nutrition Research.

[B38] Boardman B, Lewis M, Trinder P, Rajaratnam G, Croft P (2005). Use of community pharmacies: a population-based survey. Journal of Public Health.

[B39] Wazaify M, Shields E, Hughes CM, McElnay JC (2005). Societal perspectives on over-the-counter (OTC) medicines. Family Practice.

[B40] Bradley CP, Riaz A, Tobias RS, Kenkre JE, Dassu DY (1998). Patient attitudes to over-the-counter drugs and possible professional responses to self-medication. Family Practice.

[B41] Urquhart G, Sinclair HK, Hannaford PC (2004). The use of non-prescription medicines by general practitioner attendees. Pharmacoepidemiology and Drug Safety.

[B42] McElnay JC, McCallion CR (1996). Non-prescription drug use by elderly patients. The International Journal of Pharmacy Practice.

[B43] Payne K, Ryan-Woolley BM, Noyce PR (1997). Factors influencing consumers to purchase deregulated medicines over the counter. The Pharmaceutical Journal.

[B44] Kayne S, Beattie N, Reeves A (1999). Survey of buyers of over-the-counter homeopathic medicines. The Pharmaceutical Journal.

[B45] Reid S (2002). A survey of the use of over-the-counter homeopathic medicines purchased in health stores in central Manchester. Homeopathy.

[B46] Besley T, Hall J, Preston I (1999). The demand for private health insurance: do waiting lists matter?. Journal of Public Economics.

[B47] King D, Mossialos E (2005). The Determinants of Private Medical Insurance Prevalence in England, 1997–2000. Health Services Research.

[B48] Propper C, Rees H, Green K (2001). The demand for private medical insurance in the UK: a cohort analysis. The Economic Journal.

[B49] Propper C (2000). The demand for private health care in the UK. Journal of Health Economics.

[B50] McGrath C, Moles D, Bedi R (1999). Who Uses Independent Dental Services? Findings from a National Survey. Primary Dental Care.

[B51] Mulvaney C, Coupland C, Wilson A, Hammersley V, Dyas J, Carlisle R (2005). Does increased use of private health care reduce the demand for NHS care? A prospective survey of General Practice referrals. Journal of Public Health.

[B52] McManus RJ, Ryan A, Greenfield S, Pattison HM, Clifford S, Marriott J, Wilson S (2007). Self measurement of blood pressure: a community survey. Journal of Human Hypertension.

[B53] Wilson S, Ryan AV, Greenfield SM, Clifford SC, Holder RL, Pattison HM, Fitzmaurice DA, McManus RJ (2008). Self-testing for cancer: a community survey. BMC Cancer.

[B54] Cappuccio F, Cook G, Atkinson RW, Strazzullo P (1997). Prevalence, detection, and management of cardiovascular risk factors in different ethnic groups in south London. Heart.

[B55] Furnham A (2000). Attitudes towards homoeopathy in particular and beliefs about complementary medicines in general. Psychology, Health and Medicine.

[B56] Furnham A, Lovett J (2001). Predicting the use of complementary medicine: A Test of the Theories of Reasoned Action and Planned Behavior. Journal of Applied Social Psychology.

[B57] Trinder P, Rajaratnam G, Lewis M, Croft P (2003). Prophylactic aspirin use in the adult general population. Journal of Public Health Medicine.

