# Supplementary material for: Factors associated with self-care activities among adults in the United Kingdom: a systematic review
Source: BMC Public Health. 2009 Apr 5;9:96. doi: 10.1186/1471-2458-9-96 (PMC2674604; doi:10.1186/1471-2458-9-96)
Supplement: Additional file 1 — Box 1. Proforma based on tools from the Critical Appraisal Skills Programme [4,5] that was used to assess the quality of eligible quantitative studies. [file 1471-2458-9-96-S1.doc]

**Box 1:** Proforma based on tools from the Critical Appraisal Skills Programme [4, 5] that was used to assess the quality of eligible quantitative studies.

|  | Lead author and year |  |
| --- | --- | --- |
|  | Are the results valid? |  |
| **1** | Did the study addressed a clearly focused issue? |  |
| **2** | Did the authors use an appropriate method to answer the question? |  |
| **3** | Was the study population recruited in an acceptable way?  Think about selection bias e.g. representative, clear exclusion and inclusion criteria. |  |
| **4** | Was the exposure accurately measured to minimise bias? |  |
| **5** | Was the outcome accurately measured to minimise bias? |  |
| **6** | Have the authors identified all important confounding factors?  Have they taken account of the confounding factors in the design and / or analysis? |  |
| **7** | Was the analysis appropriate? |  |
| **8** | Was the study population recruited in an acceptable way?  Think about selection bias e.g. non response. |  |
|  | What are the results? |  |
| **9** | What are the results and how precise are they? |  |
| **10** | Do you believe the results? |  |
|  | **Total (out of 10)** |  |
