# Supplementary material for: Factors associated with self-care activities among adults in the United Kingdom: a systematic review
Source: BMC Public Health. 2009 Apr 5;9:96. doi: 10.1186/1471-2458-9-96 (PMC2674604; doi:10.1186/1471-2458-9-96)
Supplement: Additional file 2 — Box 2. Proforma based on tools from the Critical Appraisal Skills Programme [4,5] that was used to assess the quality of eligible qualitative studies. [file 1471-2458-9-96-S2.doc]

**Box 2:** Proforma based on tools from the Critical Appraisal Skills Programme [4, 5] that was used to assess the quality of eligible qualitative studies.

|  | Lead author and year |  |
| --- | --- | --- |
| **1** | Was there a clear statement of the aims of the research? |  |
| **2** | Is a qualitative methodology appropriate?  Think about whether the research aims to interpret or illuminate actions or subjective experiences of participants. |  |
| **3** | Was the research design appropriate to address the aims of the research?  Think about whether the researcher has justified this. |  |
| **4** | Was the recruitment strategy appropriate to the aims of the research?  Think about how people were selected and whether there is discussion of non-participation. |  |
| **5** | Were the data collected in a way that addressed the research issue?  Think about the setting, the exact method, use of topic guide, how data were recorded, saturation. |  |
| **6** | Has the relationship between researcher and participants been adequately considered? |  |
| **7** | Have ethical issues been taken into consideration? |  |
| **8** | Was the data analysis sufficiently rigorous?  Think about potential bias i.e. selection of data for presentation. |  |
| **9** | Is there a clear statement of the findings?  And think about credibility i.e. triangulation, respondent validation, more than one analyst. |  |
| **10** | How valuable is the research? |  |
|  | **Total (out of 10)** |  |
