# Supplementary material for: Factors associated with self-care activities among adults in the United Kingdom: a systematic review
Source: BMC Public Health. 2009 Apr 5;9:96. doi: 10.1186/1471-2458-9-96 (PMC2674604; doi:10.1186/1471-2458-9-96)
Supplement: Additional file 3 — Table 3. Summary of eligible studies related to use of complementary and alternative therapy (CAM). [file 1471-2458-9-96-S3.doc]

**Table 3:** Summary of eligible studies related to use of complementary and alternative therapy (CAM).

| **Year** | **Design** | **Population** | **Period** | **Exposure** | **Denominator** | **Numerator (response rate)** | **Results^a^** | **Quality** |
| --- | --- | --- | --- | --- | --- | --- | --- | --- |
| 2001 [6] | Questionnaire survey | Adults from health authority populations | 1998 | Visited CAM practitioner in last 12 months | 5010 questionnaires distributed  269 undelivered, leaving 4741 | 2853 returned but 107 blank and 78 completed by wrong person  59% (2669 / 4556) response rate given | *Unadjusted chi-squared analysis:*   - More likely in women (12.5%, 10.7-14.3) than men (8.8%, 7.3-10.3) (p < 0.01) - Significant difference by age (p < 0.001): 11.0% 18-44 (9.3-12.8), 12.9% 45-64 (10.6-15.2), 9.6% 65-74 (6.1-13.0), 4.2% > 75 (1.5 to 6.5)   *Descriptive analysis:*   - Usually for musculoskeletal problems (71%) and paid for by patients (79%) | 7.5 |
| 2003 & 2003 [7 & 13] | Questionnaire survey | Patients attending practices selected to represent range of deprivation and rurality, list size, and CAM provision | 2000 | Use of CAM therapies or remedies | 1987 received questionnaires  OR  2032 questionnaires distributed and 32 undelivered, leaving 2000 | 1523 responded  348 incomplete  Leaves 1175 but 1174 analysed  OR  1198 responses received but 24 ineligible, leaving 1174  59% response rate quoted in both papers | *Descriptive analysis:*   - NHS prescription or referral for only 13% who had used CAM in the last month   *Chi-squared analysis (unclear if adjusted or not):*   - Use (unclear if lifetime or concurrent) fell with increasing age (p < 0.005), lower educational attainment (p < 0.005) and lower household income (p < 0.005) - Use more likely in women than men (p < 0.005)   *Logistic regression analysis including factors significant in univariate analysis (education excluded as missing data):*   - - Lifetime use more likely: - Female (odds ratio (OR) 1.6, 1.19-2.17) - All age groups relative to ≥ 70 (e.g. OR 3.6 for 30-49, 2.40-5.34) - CAM provided by GP relative to not (OR 1.5, 1.07-2.04) - OTC use in past month relative to not (e.g. OR 4.8 for 1-2, 3.40-6.73)   - Concurrent use more likely: - OTC use in past month relative to not (e.g. OR 5.7 for 1-2, 4.14-7.72) - 30-49 (OR 1.9, 1.22-2.86) and 50-69 (OR 1.8, 1.20-2.74) relative to ≥ 70 | 6.5 |
| 2002 [8] | Questionnaire survey | Medical students | 1999 | Used CAM | 211 students | 150 (71%) returned  143 completed all questions | *Unadjusted analysis:*   - More likely to have used CAM if family member already used it (P < 0.001) | 7.5 |
| 2002 & 2003 [9 & 10] | Questionnaire and interview survey | Patients aged over 60 of CAM practitioners taking part in separate survey | Not given | Used CAM | 400 questionnaires distributed | 144 (36%) completed.  20 interviews but no response rate given | *Descriptive and qualitative analysis:*   - Reasons for adoption:   - Most often (36%) introduced by friend   - Inadequacies of orthodox medicine (OM) e.g. rushed appointments, over reliance on drugs and their side effects, waiting times, clinicians’ lack of care and attention and lack of success in solving health problems   - Attractions of CAM e.g. individual, holistic, natural, convenient, more sensitivity, interest and time from therapist | 4.5 |
| 2002 & 2004 [11 & 12] | Questionnaire survey | Adults aged 18-64 registered with general practices | 1997 | Consulted CAM practitioner in past 3 months  OR  Consulted osteopath / chiropractor or physiotherapist past 3 months | 14868 questionnaires distributed | 8889 eligible people completed questionnaires  64% response rate quoted after adjustment for returns from inappropriate or deceased addressees | *Regression analysis with age, sex, social class, chronic illness type, GP visits:*   - Predictors of visiting CAM practitioner: - Long-standing illness (OR 2.07, 1.73-2.49) - Non-manual social class (OR 2.00, 1.63-2.45) - Being female (OR 1.60, 1.33-1.92) - High GP use (OR 1.32, 1.09-1.58)   *Regression analysis adjusted for back pain, age, sex and social class:*   - Predictors of osteopathic / chiropractic consultation: back pain (OR 5.11, 4.05-6.44), non-manual social class (OR 2.10, 1.58-2.78), non-smoker (OR 1.50, 1.12-2.03), > 30 minutes exercise per week (OR 1.48, 1.16-1.90), being female (OR 1.26, 1.00-1.60), age (p = 0.013, 34-49 most likely to consult) - Predictors of physiotherapist consultation: back pain (OR 2.73, 2.15-3.48), non-manual social class (OR 1.76, 1.35-2.29), wants to take more exercise (OR 1.55, 1.15-2.08)   *Additionally in univariate chi-squared analyses:*   - Predictors of osteopathic / chiropractic consultation: drinks alcohol relative to not (p = 0.016), wants input to NHS decisions (p < 0.001), worries about global environment (p = 0.002), wants to do more exercise (p = 0.005) - Predictors of physiotherapist consultation were: signed healthcare petition (p = 0.047), wants input to NHS decisions (p = 0.005), worries about global environment (p = 0.025) | 8.5 |
| 2006 [14] | Questionnaire survey | Patients seeing acupuncturists | 2002 | Consulting acupuncturist | Not given | 638 (33%) of acupuncturists agreed to participate  9408 questionnaires completed but no response rate given | *Descriptive analysis*   - 74% female (p < 0.001) compared with 51% female in 2001 Census - Relatively higher proportions in 35-64 age range than 2001 Census - Only 10% advised to consult by NHS professional: most common pathways were self-referral (40%) or friend’s or relative’s recommendation (34%) | 6.0 |
| 2001 [15 & 54] | Questionnaire survey | Patients aged 40-59 years registered with GPs | 1994 to 1996 | Patients taking alternative medicines not prescribed by a doctor | 3606 invitations distributed  952 undelivered, leaving 2654 | 1695 responded (64% response rate given)  117 excluded, but 1577 in analyses | *Regression analysis adjusted for age, sex, ethnic group and social class:*   - Use more likely in: - Black African than South Asians (OR 1.66, 1.07-2.59) or people of white ethnic origin (OR 1.78, 1.07-2.94) - Females (OR 2.09, 1.45-3.00) - Use less likely in social class IV & V (OR 0.53, 0.31-0.90) than I & II | 7.5 |
| 2005 [16] | Interview study | Adults visiting CAM practices | Not given | Recent use of, and commitment to CAM | Not given | 11 interviews but no response rate given | *Qualitative analysis:*   - Main themes: - Initially got CAM to address specific problems - Dissatisfaction with OM - Holistic approach of CAM - CAM as natural and traditional - Side effects of OM - Limited effectiveness of OM - Empowering emphasis on one’s own healing capacity | 7.5 |
| 2004  [17] | Questionnaire and qualitative survey | Patients treated at NHS CAM clinic | Not given | Patient at clinic | 327 questionnaires distributed | 237 (72%) returned  86% recorded qualitative statements | *Descriptive analysis*   - Most were female (71%) - Highest proportion (about one quarter) treated in study period were 35-44   *Qualitative analysis*   - OM was not working for some patients and / or they wanted to reduce it’s risks - Symptom relief was the dominant theme and patients wanted to find ways to cope with their chronic problems - Patients expressed a desire for a holistic approach | 6.0 |
| 1993 [18] | Questionnaire and interview survey | Patients registered with general practice | Not given | Tried alternative medicine in past 10 years | 372 questionnaires distributed | 233 (63%) completed  20 interviews with registered patients known to be users of alternative treatments but no response rate given | *Unadjusted descriptive analysis:*   - 46% of women and 34% of men had used alternative medicine - Peak use at 45 - 69% of users (49% non-users) had seen GP with severe or chronic condition   *Log linear analysis:*   - Lower GP attendance rate among non-users than users (G^2^  = 46.67, 2 df)   *Interview analysis:*   - OM entails greater risk than mobilising body’s natural healing ability - Therapist’s time and attention most valued aspect - Widely accepted that NHS cannot give same attention | 5.0 |
| 2006 [19] | Questionnaire and interview survey | Students at London School of Pharmacy | 2004 | CAM use | 447 students approached | 264 (59%) completed | *Unadjusted chi-squared analysis:*   - - CAM not part of tradition of white students (not significant as small numbers) | 5.5 |
| 1996 [20] | Case note review | Treated during survey period | 1992 | Being treated by practitioner on the register of traditional Chinese medicine | 146 practitioners approached | 94 (64%) responded  62 willing but only 24 eligible, 6 withdrew and one excluded, leaving 17 with 714 active patients | *Descriptive analysis:*   - 69% female and 31% male with median age of 45 and 46 - 68% of 552 with duration recorded had symptoms for > one year - 77% of 492 with occupation recorded considered to have disposable income - 79% of 323 with referral recorded were referred via personal recommendation | 6.0 |
| 1994 [21] | Questionnaire survey | Patients visiting GP or alternative practitioner | Not given | Attended alternative therapy clinic or health centre | 200 questionnaires distributed to health centres  200 questionnaires distributed to alternative therapy clinics | 160 included: 80 from alternative therapy clinics, and 80 from health centres  48% response rate quoted with no difference between health centres and alternative therapy clinics | *Unclear if analysis adjusted or not:*   - Alternative therapy patients more likely than health centre patients to: - Disagree that only need to see alternative practitioner when ill (p < 0.001) - Disagree that treatment should concentrate on symptoms rather than the whole person (p < 0.001) - Be more health conscious and aware (p < 0.01 for 10 of 14 questions). - Have higher knowledge score (p < 0.001) - Have lower general threat to health (diseases not controllable by anyone) score (p < 0.001) - Have lower provider control (doctor can control health) score (p < 0.001) | 4.5 |
| 2000 [22] | Questionnaire survey | Recruited from university panel and by market research agency | Not given | How many complementary therapies people had tried | Not given | 159 participants, 80 from market research agency  98% response rate quoted | *Regression analysis including sex, smoker, religious, vegetarian, health, attitudes to medicine, heard of ways of telling future, tried ways of telling future, think ways of telling future works, think ways of telling future effective:*   - Number of complementary therapies tried predicted by number of ways of predicting the future they had tried (p < 0.001) | 4.5 |
| 2002 [23] | Questionnaire survey | People from register of eligible to vote | 1999 | Used at least one type of CAM | 800 questionnaires distributed | 432 (54%) completed | *Descriptive analysis:*   - 67% of people who gave details of CAM use were women and highest proportion (46%) aged 30-49 | 6.0 |
| 2000 [24] | Questionnaire survey | Adults using random-digit telephone dialling as part of regular weekly Omnibus survey | 1999 | Used CAM in previous year | Not given | 1204 interviewed but no response rate given | *Descriptive analysis*   - Use of in past year higher in: - Females (24%) than males (17%) - 35-64 years (26%) than 25-34 (20%) or 65+ (11%) - Social class AB (25%) than C1 (23%), C2 (19%) or DE (16%) - People who were working (23%) than who were not working (17%) - Most commonly (25%) because it helps or relieves the illness or condition | 6.0 |
| 1995 [25] | Questionnaire survey | Patients at outpatients, GP surgeries, and acupuncture and shiatsu clinics | Not given | At acupuncture or shiatsu clinic (CAM)  At GP surgery or outpatients and had never seen CAM practitioner (OM only) | 200 questionnaires distributed at acupuncture and shiatsu clinics and GP surgeries  100 questionnaires distributed at outpatients | 187 completed:  31 from GP surgeries (47% response rate quoted)  69 from acupuncture (67% response rate quoted) and shiatsu clinics (58% response rates quoted)  87 from outpatients (no response rate given) | *One way analysis of variance:*   - Compared to OM only group, CAM group more likely to be: female (p < 0.05), younger (p < 0.01), more left wing (p < 0.01), higher occupational status (p < 0.05), less religious (p < 0.01)   *One way analysis of variance adjusted for age, sex, political beliefs, occupational status and religion:*   - Compared to OM only group, CAM group believed these factors were more important: psychological (p < 0.05), environmental (p < 0.05), emotional well-being (p < 0.05), self-medication (p < 0.01), medical treatment (p < 0.01) | 7.5 |
| 2003  [26] | Questionnaire survey | Outpatients at the Royal London Homeopathic Hospital | 1997 | Three consultations | 786 eligible, but 245 did not attend, missed, declined or excluded  541 questionnaires distributed | 506 returned but 7 more excluded  93% response rate given | *Descriptive analysis*   - Most were female (81%) and white (81%) - Most (79%) had asked GP for referral - Most frequent reason for seeking CAM (304 of 493) was that other treatment had not helped | 6.5 |
| 1996 [27] | Questionnaire survey | Patients waiting for treatment | Not given | Attended Royal Homeopathic Hospital, acupuncture centre, or British School of Osteopathy | Not given | 268 patients: 87 homeopathy, 92 acupuncture, and 89 osteopathy  78% average response rate for three groups quoted | *Unadjusted descriptive analysis:*   - Mostly females: 60% osteopathy, 81% homeopathy, 84% acupuncture - Reason for treatment with mean rating 4 or more out of 5 in all groups:   - OM ineffective for problem   - CAM would be more effective   - CAM allows active role   - Value emphasis on whole person   - Relaxed after CAM   - Practitioner’s explanation makes sense | 6.0 |
| 2004 [28] | Questionnaire survey | Adult respondents to Omnibus survey | 2001 | Used practitioner to receive CAM in past 12 months | 2761 eligible addresses | 1794 (65%) respondents | *Unadjusted descriptive analysis:*   - 52% not told GPs of visits to CAM practitioners - Used less in north (4.3%, 2.8-6.5) than all England (10.0%, 8.6-11.7) - Proportions using CAM varied with age (p = 0.012): highest (14%) 45-54 - Use more likely if: - Income ≥ £15.6k (14.4%, 11.6-17.7) than lower (7.8%, 6.4-9.5) - Non-manual (14.1%, 12.0-16.4) than manual social class (4.9%, 3.6-6.8) - Full-time education to ≥ 19 (19.7%, 15.9-24.1) than < 19 (7.7%, 6.4-9.3) | 7.5 |
| 1993 [29] | Questionnaire survey | Patients visiting general practice, or Royal Homeopathic Hospital | Not given | Being a patient | 100 questionnaires distributed to each setting | 160 included:  80 completed by homeopathic patients  90 completed by general practice patients but last 10 discarded | *Unadjusted analysis:*   - - Homeopathy patients more likely to be older (mainly 41-50 compared to 31-40, p < 0.05) and have higher incomes (p < 0.05) than GP patients   *Multivariate analysis of covariance adjusted for age and income:*   - - Homeopathy more likely than GP patients to: - Believe they will get asthma (p < 0.001) - Believe they will get sleeplessness (p < 0.05) - Believe good diet (p <0.05), relaxation (p < 0.001), sleeping (p < 0.01), meditation (p < 0.001), less drinking (p < 0.01), less smoking (p < 0.05), less stress (p < 0.001) prevents illness - Believe in treating whole person (p < 0.001) - Believe that body can heal itself (p < 0.01) - Have tried acupuncture (p < 0.001) and osteopathy (p < 0.001) - Be dissatisfied with OM (p < 0.001) - Think they will live longer (p < 0.05) - Have higher Langner mental health scores (p < 0.001) - Believe they have greater self-control over health (p < 0.05) - Homeopathy less likely than GP patients to: - Like to leave their health in others’ hands (p < 0.01) - Be satisfied with last visit to practitioner (p < 0.001) - Take notice of TV or radio health recommendations (p value unclear) | 7.0 |
| 1995 [30] | Questionnaire survey | Patients attending CAM and OM practitioners | Not given | Attended Royal Homeopathic Hospital, acupuncture centre, British School of Osteopathy, or general practice | Not given | 256 patients: 76 homeopathy, 57 acupuncture, 65 osteopathy, and 58 general practice  Response rate of 60% given for general practice patients and over 70% for other groups | *One way analysis of variance:*   - Significant differences between groups (p < 0.05) with GP patients: least likely to be consulting another practitioner, oldest, shortest illness, least likely to have had serious illness in last 5 years, least likely to have chronic illness - Significant differences between groups (p< 0.001) with GP patients least agreeing, although all agreed, that CAM practitioners are more sympathetic and sensitive, better at explaining, and have more time to listen   *Analysis of covariance adjusted for age, number of children, significant medical history variables:*   - GP patients have least healthy lifestyles (p < 0.05) and place least importance on a healthy mind (p < 0.05) - Significant differences between groups (p < 0.05) with GP patients: - Most satisfied with GPs, confident about prescribed drugs, faith in medical science, support for science - Least likely to read about health or be concerned about medicine’s harmful effects or global environment | 6.0 |
| 1997 [31] | Questionnaire survey | New patients of CAM practitioner, or GP patients with symptoms for more than 7 days | 1994 to 1995 | Consulted practitioner | 68 CAM patients  659 GP patients but 441 ineligible, leaving 218 | 254 initial questionnaires completed: 46 complementary practitioner and 208 GP  191 follow-up questionnaires completed: 34 CAM practitioner and 157 GP | *Descriptive analysis*   - 73% of CAM patients and 50% of GP patients had symptoms for > 12 weeks   *Chi-squared analysis:*   - CAM patients more likely to have had longer education (p = 0.003)   *Mann-Whitney analysis:*   - CAM patients have lower bodily pain scores (p = 0.013) | 5.5 |
| 1995 [32] | Questionnaire survey | Patients attending CAM and OM practitioners | Not given | Attended Royal Homeopathic Hospital, acupuncture centre, British School of Osteopathy, or general practice | Not given | 216 patients: 73 homeopathy, 47 acupuncture, 46 osteopathy, 50 general practice  60% initial response rate given with no difference between groups | *One-way analysis of variance:*   - Significant difference between groups for length of illness (p < 0.005): GP patients had shortest illness | 7.0 |
| 1996 [33] | Questionnaire survey | Random sample of residents on a population health register | 1993 | Used CAM | 500 questionnaires distributed  10 undelivered, leaving 490 | 341 (70%) completed | *Descriptive analysis:*   - Most often (58%) chosen because of friend’s or colleague’s recommendation - Next highest proportion (28%) indicated that a doctor or health professional had either referred them or recommended CAM | 7.0 |
| 2000 [55] | Questionnaire survey | Recruited from university panel and by market research agency | Not given | Ever tried CAM | Not given | 430 participants, 70% from market research agency  Response rates given as 95% for market research agency and 92% for university panel | *Regression analyses adjusted for number of therapies heard of, and used, sex, age, religious / political beliefs, history of serious illness, comparative / current health:*   - Less well disposed to homeopathy if tried fewer therapies (p < 0.05) - More well disposed to homeopathy if tried more therapies (p < 0.01) | 6.0 |
| 2001 [56] | Questionnaire survey | People approached at various locations | Not given | Homeopathy use assessed at 4 weeks | 500 questionnaires distributed | 343 (69%) returned  139 contacted at 4 weeks | *Regression analysis including sex, intention to use homeopathy, perceived behavioural control, self-efficacy, prior use of homeopathy:*   - Use associated with past use (p < 0.01) and intention to use (p < 0.001). | 7.0 |

^a^ This table only shows results that are significant or reported as key findings rather than the results of all variables tested in analyses.
