# Supplementary material for: Factors associated with self-care activities among adults in the United Kingdom: a systematic review
Source: BMC Public Health. 2009 Apr 5;9:96. doi: 10.1186/1471-2458-9-96 (PMC2674604; doi:10.1186/1471-2458-9-96)
Supplement: Additional file 4 — Table 4. Summary of eligible studies related to use of over-the-counter (OTC) medicine [file 1471-2458-9-96-S4.doc]

**Table 4:** Summary of eligible studies related to use of over-the-counter (OTC) medicine.

| **Year** | **Design** | **Population** | **Period** | **Exposure** | **Denominator** | **Numerator (response rate)** | **Results^a^** | **Quality** |
| --- | --- | --- | --- | --- | --- | --- | --- | --- |
| 2001 [6] | Questionnaire survey | Adults from health authority populations | 1998 | Purchased CAM OTC in past 12 months | 5010 questionnaires distributed  269 undelivered, leaving 4741 | 2853 returned but 107 blank and 78 completed by wrong person  59% (2669 / 4556) response rate given | *Unadjusted chi-squared analysis:*   - More likely in women (32.6%, 30.0-35.1) than men (12.0%, 10.2-13.8) (p < 0.001) - Significant difference by age (p < 0.001): 23.5% 18-44 (21.1-25.9), 24.6% 45-64 (21.6-27.5), 16.8% 65-74 (12.4-21.2), 15.5% > 75 (10.9-20.2) | 7.5 |
| 2005 [34] | Questionnaire survey | Adults on electoral roll | 2002 | Used OTC analgesics in last two weeks | 3000 questionnaires distributed  292 people had died, moved or were excluded, leaving 2708 | 1501 (55%) completed | *Stepwise regression analysis with significant factors from chi-squared analysis:*   - - Predictors of use: - < 60 relative to ≥ 60 (odds ratio (OR) 1.52, 1.05-2.20) - Female relative to male (OR 1.70, 1.33-2.18) - O’levels or better relative to up to O’level (OR 1.47, 1.12-1.94) - Poor / fair (OR 2.01, 1.20-3.36) and good / very good (OR 1.94, 1.28-2.94) relative to excellent health - Pays relative to not pays prescription fees (OR 1.55, 1.10-2.13) - Not relative to using prescription analgesics (OR 2.17, 1.49-3.14)   *Additionally in unadjusted chi-squared analysis:*   - - Use of non-prescription analgesics more likely if: - Married relative to other status (OR 1.32, 1.07-1.64) - Drinks alcohol relative to does not (OR 1.84, 1.41-2.39) - More relative to less affluent (p ≤ 0.01 for trend, OR 2.69 for category 1 relative to 7, 1.45-5.01) | 9.5 |
| 2002 [35] | Focus group and interview study | Focus groups: 16-24s: job-seekers, students, mothers, people with asthma  Interviews: people asking for advice about ailment or buying treatment | 1997 to 1998 | Management of minor ailments | Not given | 48 focus group participants (response rate not given)  76 structured interviews (74% response rate)  9 in-depth interviews (90% response rate) | *Unadjusted descriptive and qualitative analysis:*   - Decision to ask for medicine most commonly (38%) influenced by mother - Schemas to manage minor ailments drawn up from experiences - Combine salient information from adverts with past experience | 6.5 |
| 2006 [36] | Interview study | Women attending yoga groups, therapy centre and women’s book group | Not given | Used herbal medicine in past 12 months | 70 preliminary questionnaires distributed | 18 herbal medicine users responded and all agreed to be interviewed | *Qualitative analysis:*   - Motivations: - Perceived advantages: most often (15 of 18) personal control - Disadvantages of conventional care: most often (6 of 14) that doctors are unable to help with some problems - Disadvantages of conventional medicines: most often (12 of 12) that they are chemical | 7.0 |
| 2004 [37] | Questionnaire survey | Adults on health authority general practice register | 2001 | Usually take herbal supplements | 21923 questionnaires distributed | 15465 (71%) completed | *Regression analysis adjusted for age, sex, housing tenure:*   - More likely in: - 45-64 than 18-44 (OR 1.45, 1.34-1.67) - Women (OR 3.11, 2.79-3.48) - White than non-white (OR 2.45, 1.75-3.44) - Active than sedentary (OR 1.29, 1.16-1.43) - Possible than unlikely psychiatric morbidity (OR 1.21, 1.08-1.34) - On than not on prescribed medication (OR 1.13, 1.02-1.26)   - Less likely in > 75 than 18-44 (OR 0.88, 0.82-0.94)   *Plus in regression analysis adjusted for age, sex:*   - - More likely in private than rented housing (OR 1.74, 1.55-1.99)   *Additionally in unadjusted analysis:*   - - More likely in non-smoker than smoker (OR 1.14, 1.02-1.26) | 9.0 |
| 2005 [38 & 57] | Questionnaire survey | Adults 35 years or older on health authority register | 2000 | Purchased OTC medicine in previous month | 10000 questionnaires distributed  9469 excluding deaths and departures | 6322 (67%) completed | *Unadjusted chi-squared analysis:*   - - Less likely among people < 60 who were exempt from charges than those who would have to pay (OR 0.75, 0.6-0.9)   *Regression analysis adjusted for age, sex, social class, health, exercise, smoking, perceived vascular risk:*   - - More likely: - Female (OR 1.86, 1.66-2.08) - Poor (e.g. OR 1.78, 1.32-2.41) and fair or good than excellent health - Perceive vascular risk than no risk (OR 1.21, 1.06-1.39)   - - Collecting prescription medicine (OR 2.02, 1.79-2.28)   - Less likely:     - All age groups than 35-44 (e.g. OR for 65-74 0.47, 0.39-0.57)     - Intermediate or routine / manual (e.g. OR 0.77, 0.67-0.88) than professional / managerial occupation | 8.5 |
| 2005 [39] | Questionnaire survey | Members of the public in shopping centres | 2002 | Purchased non-prescription medicines | Not given | 1000 participated (response rate not given) | *Unadjusted chi-squared analysis:*   - More likely to buy weekly or monthly than less often if: - Female (36%) than male (27%) (p < 0.05) - ≤ 60 (36%) than > 60 (21%) (p < 0.001) - Pays for prescriptions compared with exempt (p < 0.05) | 6.0 |
| 1998 [40] | Questionnaire survey | Adults attending general practices | 1995 | Used OTC remedies regularly | 3030 questionnaires distributed | 2765 (91%) completed  141 excluded, leaving 2624 | *Unadjusted chi-squared analysis:*   - - More frequent in females than males (p < 0.01) and social class I-IIINM than IIIM-V (p < 0.01)   - Varied significantly with age (p < 0.01) and highest (31%) in 45-64 | 8.5 |
| 2004 [41] | Questionnaire survey | People attending general practices | 1999 | Used non-prescription medicine in last 7 days | General practices purposively chosen  461 eligible patients approached | 427 (93%) completed pre-consultation questionnaire  305 completed pre- and post-consultation questionnaires (71% response rate given) | *Stepwise regression analysis including age, sex, deprivation, prescription charges, used prescribed medicine in last seven days, and practice:*   - Use more likely in < 60 than > 60 (OR 1.85, 1.13-3.02)   *Additionally in univariate chi-squared analysis:*   - Use more likely in not than exempt from charges (OR 1.71, 1.07-2.73) - Use less likely in least than most affluent group (OR 0.28, 0.09-0.90) | 7.0 |
| 1996 [42] | Questionnaire survey | Adults ≥ 65 at community pharmacies in Northern Ireland | 1993 & 1994 | Purchased non-prescription drugs | Not given for pharmacies or patients | 515 completed  Response rate not given for pharmacies or patients | *Unadjusted descriptive analysis:*   - - Most common reason (about 19%) that symptoms not severe enough for doctor | 4.0 |
| 1997 [43] | Questionnaire survey | Customers at pharmacies | 1996 | Bought H2 anatagonists for dyspepsia, aciclovir cream for cold sores, imidazoles for thrush, nasal spray for hay fever, or got them on private prescription | 311 pharmacies  3000 questionnaires distributed | 679 (23%) returned  628 eligible | *Regression analysis including 18 (of initial 23) significant (p < 0.05) variables from chi-squared analysis (no significance levels given for regression analysis):*   - Top predictors of OTC purchase: - Preference - Knowledge of availability - Liability for prescription charge - Not taking prescription medicine | 4.5 |
| 1999 [44] | Questionnaire survey | Customers at pharmacies selling homeopathic remedies | 1996 to 1998 | Purchased homeopathic medicines | 120 pharmacies approached  1090 questionnaires distributed | 109 (91%) pharmacies participated  417 questionnaires returned  10 spoiled, leaving 407 (37%) | *Unadjusted descriptive analysis:*   - 78% of 404 who gave their gender were female - Highest proportion (30%) of 404 who gave their age were 46-60 - Most frequent prompt (67%) was that they always use them | 5.0 |
| 2002 [45] | Questionnaire survey | People who had just purchased OTC homeopathic remedies in health food shops | 2000 to 2001 | Purchased OTC homeopathy | 3 shops approached  75 people approached | All shops participated  2 people refused, leaving 73 | *Unadjusted descriptive analysis:*   - 62 of 75 people approached were female - Highest proportion of respondents (30%) were 36-45 years - Most common reason (72% strongly agree) was more natural - First purchase most often (45%) prompted by friend or family member | 5.0 |

^a^ This table only shows results that are significant or reported as key findings rather than the results of all variables tested in analyses.
