# Supplementary material for: Factors associated with self-care activities among adults in the United Kingdom: a systematic review
Source: BMC Public Health. 2009 Apr 5;9:96. doi: 10.1186/1471-2458-9-96 (PMC2674604; doi:10.1186/1471-2458-9-96)
Supplement: Additional file 5 — Table 5. Summary of eligible studies related to use of the private sector. [file 1471-2458-9-96-S5.doc]

**Table 5:** Summary of eligible studies related to use of the private sector.

| **Year** | **Design** | **Population** | Period | **Exposure** | **Denominator** | **Numerator (response rate)** | **Results^a^** | **Quality** |
| --- | --- | --- | --- | --- | --- | --- | --- | --- |
| 1999 [46] | Estimation of model of demand for individually purchased private health insurance | British Social Attitudes survey respondents | 1986 to 1991 | Purchased private health insurance | Not given | Annual survey of about 3000 (no response rate given) | *Unadjusted descriptive analysis (no significance levels):*   - 50-65 highest proportion with insurance (27% v. 22% with no insurance) - 66% with insurance and 37% with no insurance have income > £15k - 13% with insurance and 7% with no insurance have degree - 88% with insurance and 66% with no insurance were home owners - 59% with insurance and 33% with no insurance supported the right   *Estimates of demand for individually purchased private health insurance using more sophisticated model including regional dummy variables:*   - - Positively associated with: - Income (z statistic = 13.4) - Educational attainment (z statistic highest for A levels = 7.2) - Age but falls > 65 years (z statistic highest for 50-65 = 7.0) - Being a home owner (z statistic = 4.8) - Long-term NHS waiting lists (z statistic = 1.8)   - Negatively associated with: - Number of adults in household (z statistic = -5.1) - Being employed in public sector (z statistic = -2.7) | 3.5 |
| 2005 [47] | Panel survey | Adults taking part in British Household Panel Survey | 1997 to 2000 | Purchased private medical insurance | Not given | Sample in 1996 included 7910 individuals  7.6%, 8.0%, 8.0% and 8.1% lost, respectively, in 1997,1998, 1999 and 2000, but increased to 8529 in 2000  No response rate given | *Random effects logistic regression analysis:*   - More likely:   - As age increases (odds ratio (OR) = 1.29 per year, p < 0.001)   - Basic qualification relative to less (OR = 8.54, p < 0.001)   - Paid work relative to not (OR = 2.13, p = 0.001)   - As income increases (OR = 1.0002 per £1 per month, p < 0.001)   - If professional or manager relative to semi-skilled, unskilled or unemployed (OR = 1.84, p = 0.005)   - Centre-right relative to other (OR = 3.44, p < 0.001)   - As supply surgeons in region increases (OR = 1.67, p = 0.041)   - As inpatient waiting time increases (OR = 1.04, p = 0.002) - Less likely if female (OR = 0.32, p < 0.001) and as outpatient waiting time increases (OR = 0.98, p = 0.02) | 7.5 |
| 2001 [48] | Cohort analysis | Family Expenditure Survey respondents | 1978 to 1996 | Purchased private medical insurance | Not given | Overall sample size of survey is 77601 (no response rate given) | *Weighted least squares regression analysis*:   - Increases with age but older less likely than younger cohorts - Positively associated with income (significance unclear) - Positively associated with number of part-time consultants and private hospitals in region (significance unclear) - Negatively associated with number of NHS beds (significance unclear) | 6.0 |
| 2000 [49] | Estimation of model of the use of health care services | British Household Panel Survey respondents | Not given | Used private medical or dental care | Not given | Annual survey of about 5000 households (no response rate given) | *Multinomial logit model of the use of public and private care:*   - Private care positively associated with: - Being employed (z statistic = 1.5) - Income (z statistic = 9.2) - Being a conservative voter (z statistic = 5.6) - Being less supportive of NHS principles (z statistic = 4.7) - Using private care last year (z statistic = 36.4) - Private care negatively associated with: - Living in rented housing (z statistic = 10.3) - Being limited in daily activities (z statistic = 2.4) - Used NHS care last year (z statistic = 21.8) | 3.5 |
| 1999 [50] | Questionnaire survey | ≥ 16 and taking part in the Omnibus survey | Not given | Used private dental care | 2668 eligible addresses  232 people uncontactable | 571 people refused  1865 (70% of 2668) participated | *Chi-squared analysis with compensation for multiple testing:*   - Income most significant determinant (p < 0.05, 46% if > £30k, 28% if £10-30k, 16% if < £10k) | 7.0 |
| 2005 [51] | Prospective survey of GP referrals | General practices in Trent Focus Collaborative Research Network | 2001 | Referral to NHS or private sector | Not given | 10 practices (no response rate given)  100263 registered patients | *Regression analysis including sex, age group, practice, specialty, deprivation:*   - Less likely as deprivation increases (OR 0.17 for most compared with least deprived quintile, 0.13-0.22) - Most likely if 45-54 (OR 2.75 relative to < 5, 1.66-4.55) - Associated with specialty and practice | 8.5 |

^a^ This table only shows results that are significant or reported as key findings rather than the results of all variables tested in analyses.
